# Neuroprotective Properties of Oleanolic Acid—Computational-Driven Molecular Research Combined with In Vitro and In Vivo Experiments

**DOI:** 10.3390/ph16091234

**Published:** 2023-08-31

**Authors:** Katarzyna Stępnik, Wirginia Kukula-Koch, Wojciech Plazinski, Magda Rybicka, Kinga Gawel

**Affiliations:** 1 Department of Physical Chemistry, Institute of Chemical Sciences, Faculty of Chemistry, Maria Curie–Sklodowska University in Lublin, Pl. M. Curie-Skłodowskiej 3, 20-031 Lublin, Poland; 2 Department of Pharmacognosy with Medicinal Plants Garden, Medical University of Lublin, ul. Chodzki 1, 20-093 Lublin, Poland; virginia.kukula@gmail.com; 3 Department of Biopharmacy, Medical University of Lublin, ul. Chodzki 4a, 20-093 Lublin, Poland; wojtek_plazinski@o2.pl; 4 Jerzy Haber Institute of Catalysis and Surface Chemistry, Polish Academy of Sciences, ul. Niezapominajek 8, 30-239 Kraków, Poland; 5 Department of Photobiology and Molecular Diagnostics, Intercollegiate Faculty of Biotechnology, University of Gdansk and Medical University of Gdansk, ul. Abrahama 58, 80-307 Gdańsk, Poland; magda.rybicka@biotech.ug.edu.pl; 6 Department of Experimental and Clinical Pharmacology, Medical University of Lublin, ul. Jaczewskiego Str. 8b, 20-090 Lublin, Poland; kingagawel@umlub.pl

**Keywords:** molecular dynamic simulations, molecular docking, neuroprotection, acetylcholinesterase, zebrafish

## Abstract

Oleanolic acid (OA), as a ubiquitous compound in the plant kingdom, is studied for both its neuroprotective and neurotoxic properties. The mechanism of acetylcholinesterase (AChE) inhibitory potential of OA is investigated using molecular dynamic simulations (MD) and docking as well as biomimetic tests. Moreover, the in vitro SH-SY5Y human neuroblastoma cells and the in vivo zebrafish model were used. The inhibitory potential towards the AChE enzyme is examined using the TLC-bioautography assay (the IC_50_ value is 9.22 μM). The CH-π interactions between the central fragment of the ligand molecule and the aromatic cluster created by the His440, Phe288, Phe290, Phe330, Phe331, Tyr121, Tyr334, Trp84, and Trp279 side chains are observed. The results of the in vitro tests using the SH-SY5Y cells indicate that the viability rate is reduced to 71.5%, 61%, and 43% at the concentrations of 100 µg/mL, 300 µg/mL, and 1000 µg/mL, respectively, after 48 h of incubation, whereas cytotoxicity against the tested cell line with the IC_50_ value is 714.32 ± 32.40 µg/mL. The in vivo tests on the zebrafish prove that there is no difference between the control and experimental groups regarding the mortality rate and morphology (*p* > 0.05).

## 1. Introduction

The increase in the average life expectancy is associated with the improvement of people’s awareness, the availability of medicine, and greater effectiveness of pharmacological treatment results in civilization diseases, including memory disorders, malignant changes, hormonal disturbances, and others. Apart from oncological problems, neurodegenerative diseases constitute a serious therapeutic challenge. Problems associated with neuronal functions are observed with increasing age. These include the deterioration of memory pathways, known as dementia [1,2].

These cognitive impairments are associated with reduced levels of acetylcholine, a neuromodulator secreted by neurons to initiate the action potential necessary for memorizing and retrieving memories. A decrease in the concentration of acetylcholine combined with an increased efficiency of enzymes that break it down prematurely, still in the synaptic cleft before connecting to an appropriate cholinergic receptor, causes the memory process to be disturbed [3].

In addition, with age, beta-amyloid plaques appear on the surface of neurons. They disturb the physiological functioning of neurons, cause inflammation on the surface of neuronal cells, and lead to an accelerated death of nerve cells [4].

Modern medicine has long been trying to find an appropriate therapy to prolong the efficiency of the central nervous system (CNS). Pharmaceuticals available on the market are able to slow down the aging process of neurons, reduce inflammatory conditions in the CNS, and increase the acetylcholine uptake by inhibiting the enzymes that break it down. The latter are the first-line drugs in the treatment of dementia and Alzheimer’s disease, which is a consequence of the rapidly progressing neurodegenerative changes in the CNS. Current therapy is able to slow down the course of the disease. However, the available drugs do not reverse their effects. Among the first-line drugs, there are substances of plant origin—the commonly used natural products include galantamine isolated from snowdrop, rivastigmine, a semi-synthetic derivative of physostigmine derived from calabar bean, as well as huperzine A from club moss (*Huperzia serrata*) and berberine from barberry species [5,6]. These plant-derived compounds, together with other synthetic drugs used in the treatment of dementia, have numerous side effects, such as nausea, apathy, low tolerance to the applied dose, short duration of action, and others. Some of them affect the activity of acetylcholinesterase (AChE), which is one of the two enzymes found in the intersynaptic cleft. Inhibition of both enzymes, AChE and the non-specific butyrylcholinesterase, is much more beneficial because it has a greater effect on the memory process [6,7].

In view of the above limitations of the existing therapy, it is necessary to search for new substances, also of plant origin, which could influence memory processes effectively and extend the independent functioning of many patients suffering from dementia.

**Figure 1:**
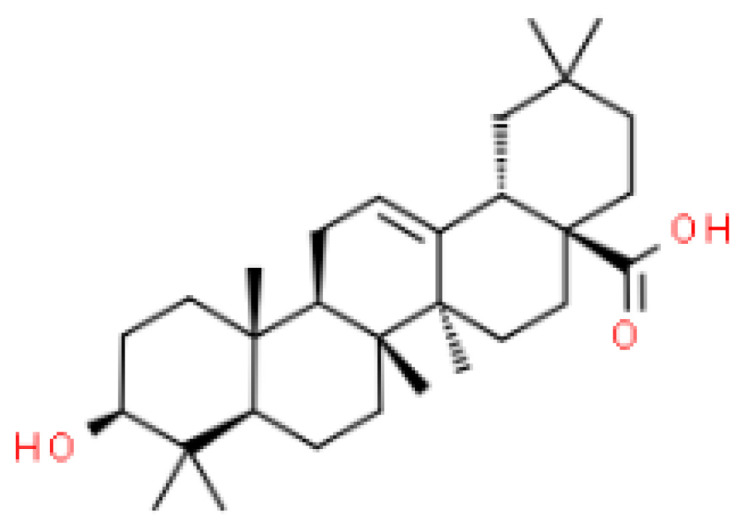
Chemical structure of oleanolic acid.

In the search for new drug candidates, the authors of this paper decided to investigate the pharmacological potential of oleanolic acid (Figure 1)—a plant-derived secondary metabolite from the group of triterpenoid saponins identified in various plant species from the following botanical families: Lamiaceae, Fabaceaae, Myrtaceae, Oleaceae, Rosaceae, Pentaphylaceae, and others [8,9]. It is important to note that OA is one of the most commonly distributed triterpenoids in nature [9], for which several isolation protocols and biosynthetic pathways have been elaborated [10,11].

Oleanolic acid, a pentacyclic triterpenoid, was used in traditional Chinese medicine as an effective drug for the treatment of hepatic disorders. More recent studies have proven its antioxidant, antitumor, weight- and cholesterol-reducing, antidiabetic, and anti-inflammatory properties [9].

Considering the above statement and the good availability of OA, the authors found it important to study its neurological potential. This manuscript aims to determine its inhibitory properties towards acetylcholinesterase enzyme, its neuroprotective properties in the tests carried out on the SH-SY5Y human neuroblastoma cell line, its toxicity that will be tested in the zebrafish model, and finally, its potential mechanism of action that will be modeled by both computational and membrane-like techniques.

## 2. Results

### 2.1. The Pharmacokinetic In Silico Studies on BBB Permeation

The BBB pharmacokinetic descriptors were calculated using the ACD/Percepta software. The following were obtained: logBB—the logarithm of blood-to-brain partition coefficient; logPS—the logarithm of permeability–surface area product; logPSFb—the brain/plasma equilibration rate; Fu—the unbound fraction in plasma; Fb—the unbound fraction in the brain (Table 1). In order to extend the interpretation of BBB parameters, there were also calculated some important physicochemical descriptors using the ACD/Percepta software: logPow—the logarithm of n-octanol/water partition coefficient; TPSA—the topological polar surface area; MW—the molecular weight.

### 2.2. Anisotropic Membrane-like Systems

In order to determine the OA permeability through the BBB, High-Performance Liquid Chromatography (HPLC) was applied using cholesterol-bound (CHOL) as well as Immobilized Artificial Membrane (IAM) and Internal Surface Reverse Phase (ISRP) anisotropic membrane-like stationary phases.

The retention data obtained from these systems allow us to calculate the logarithms of retention factors extrapolated to the pure water (logkw) based on the Soczewiński–Wachtmeister Equation (1) [10]. These values are considered to be an alternative to the logPow (n-octanol/water partition coefficient) lipophilic descriptor [11]. The values obtained from Equation (1) are presented in Table 2.

logk = logkw – sφ(1)

where logk is the logarithm of the retention factor; φ is the volume fraction of an organic modifier; s is the slope characteristic of a single solute in the given chromatographic system.

### 2.3. QSAR Analysis for BBB Permeation

Based on our previously established model (2) [12], there was obtained the logBB value. For this purpose, some physicochemical parameters, as well as excess molar refraction from the Linear Free Energy Relationship (LFER) employed by Abraham, were calculated in silico using the ACD/Percepta software (Table 3).

logBB = −0.114 − 0.098 ΔlogP + 0.278 logkw + 0.218E n = 40, R^2^CV = 78.25%, R^2^pred = 74.02%, S = 0.436 (2)

where E is the excess molar refraction and ΔlogP is the hydrogen-binding potential being the difference between the logkw lipophilic descriptor (in this case obtained from the membrane-like systems: logkw_ISRP_, logkw_IAM_, logkw_CHOL_) and the cyclohexane/water (logPcw) partition coefficients values.

**Figure 2:**
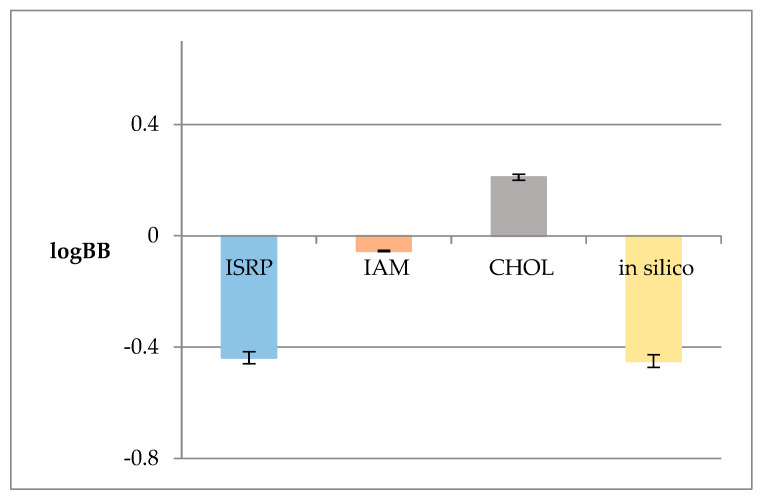
LogBB values calculated based on the QSAR model logkw values from the anisotropic membrane-like systems (ISRP, IAM, CHOL) and that calculated in silico using the ACD/Percepta software.

Since the logkw value obtained from the anisotropic membrane-like analysis is widely treated in the chromatographic practice as a lipophilicity descriptor, in this model, it was applied instead of the logPow value used in the previous one. This modification was introduced to the model to examine whether the applied membrane-like analysis proved to be effective in estimating the ability of OA to permeate through the BBB. Figure 2 shows the logBB values obtained based on Equation (2) and the lipophilic logkw values from the anisotropic membrane-like systems.

### 2.4. Acetylcholinesterase Inhibitory Activity of OA

**Figure 3:**
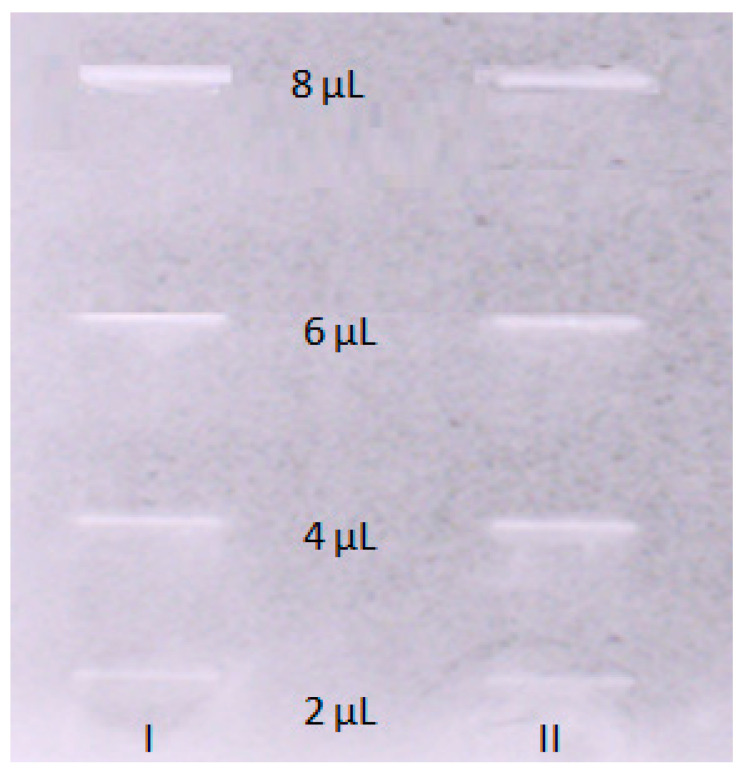
TLC bioautography results for acetylcholinesterase inhibition visualized in daylight. The TLC plate shows different concentrations of OA. I—1st; II—2nd replications.

The TLC-bioautography with the acetylcholinesterase enzyme as a spraying agent was performed to determine the AChE inhibitory properties of OA in the in vitro experiment and to find out whether the computational studies properly predicted the pharmacological properties of the compound. The interactions taking place on a TLC plate resemble those occurring in the brain. Spraying the TLC plate with a compound dispersed on its surface with an enzyme allows for direct contact of the enzyme with the compound of interest. This provides sufficient data to find out whether a compound interacts–inhibits the enzyme by competing with a substrate. The bright inhibition zones being observed on a TLC plate show an effective inhibition of the enzyme by the compound present on the TLC plate. In order to calculate the IC_50_ value, which is a measure of the compound’s strength, and demonstrate the concentration of the compound of interest that inhibits the enzyme activity by 50%, five volumes of the OA stock solution (2, 4, 6, and 8 µL) corresponding to 0.002, 0.004, 0.006, and 0.008 mg of OA on the surface of the TLC plate were introduced onto the normal phase TLC plate covered with silica gel (Merck, Darmstadt, Germany). Later, the developed TLC plate was analyzed using an imaging program that transforms the obtained greyish zones, based on their intensity, to numerical value–peak areas. The experiment was repeated three times, and the average value of the obtained peak areas corresponding to different volume injections of OA was taken for the calculation of the IC_50_ value. As a result, there was obtained the following calibration curve equation: y = 12491x − 12671 (R^2^ 0.981). Based on this equation, the IC_50_ of OA was calculated to be 9.22 μM. The respective chromatogram is presented in Figure 3. At the same time, a similar test was performed for the standard of galanthamine. The IC_50_ calculated for this alkaloid was equal to 0.044 µM.

### 2.5. Free Energy Calculations and Molecular Docking

**Figure 4:**
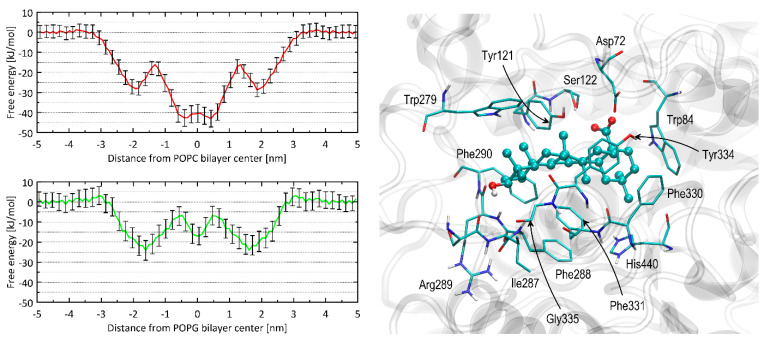
(**left**) The 1D free energy profiles accompanying the OA molecule movement through the system, including lipid bilayer. The systems contained two types of uniform bilayers: comprising POPC (red lines), or POPG (green lines). The associated error values (shown as vertical bars) were determined using bootstrapping. (**right**) The most favorable binding pose of the OA molecule interacting with AChE. The OA molecule is visualized as ball-and-stick, while amino acid residues in close proximity (within a distance of 0.38 nm) are depicted as thin sticks. Further details about the types of interactions are available in the text. The residue numbering is consistent with the PDB:3EVE record.

Atomistic molecular dynamics simulations were executed to ascertain the affinity between OA and the lipid bilayers of homogeneous composition composed of either POPC or POPG phospholipids. These bilayers, within the scope of the present study, act as simplified representations of the blood–brain barrier. The 1D free energy profiles based on a single coordinate and associated with OA permeation through the bilayer are illustrated in Figure 4.

Both calculated free energy profiles validate OA’s strong attraction to the lipid bilayer. This effect is expected due to its positive value of the logP_ow_ parameter (varying from ca. 6.5 to 8.5, depending on the source). In none of the cases, the free energy barrier associated with the immersion into the bilayer was of significant height (<5 kJ/mol). Instead, some barriers of the height of ca. 12–17 kJ/mol appear within the bilayer center. They are associated with the full immersion of whole molecules into the lipid bilayer and disrupting the polar contacts between the hydroxyl and carboxyl groups of OA with the polar head groups of phospholipids. The immersion process is associated with the free energy change of either ca. 43 kJ/mol (POPC) or 23 kJ/mol (POPG), which indicates clearly that OA has a tendency to accumulate in the lipid bilayers and the permeability rate is associated with leaving the bilayer and migrating through it rather than entering it.

When considering interactions with AChE studied using the docking simulations, OA exhibits a high value of binding energy, equal to −61.5 kJ/mol, which indicates a favorable and strong ligand–protein binding.

The results of the docking study were interpreted in terms of the mechanistic interaction pattern, which is important to understand the origin of the favorable binding energies and the mechanism of inhibition. The summary given below presents the analysis of the ligand–protein contacts that occur when the distance between any pair of corresponding atoms is smaller than the arbitrarily accepted value is 0.38 nm. The latter cutoff value is chosen particularly based on our experience in Vina-based docking and on the nature of the intermolecular interactions expected to occur in the biomolecular systems. Namely, such value allows us to spot all the polar interactions (including hydrogen bonds for which the typical donor-acceptor distance varies 0.25–0.35 nm) but also non-polar contacts characterized by larger interatomic distances (equal to ca. 0.30–0.38 nm in the case of carbon–carbon contact). For the system being presented, no additional amino-acid residues are included in the set of those interacting with the ligand upon increasing the cutoff value up to 0.45 nm.

The essential residues involved in the ligand–protein interactions identified upon accepting such a cutoff value are shown in Figure 4. It should be stressed that the reported results appeared to be reproducible when repeating the docking procedure three times. In particular, exactly the same binding energy values were obtained for the most energetically favorable configurations, and the binding arrangement of the docked ligand differed only by the marginal spatial shifts (<0.02 nm when expressed as RMSD). This is a consequence of the relatively small dimensions of the binding cavity with respect to the size of the ligand molecule; due to the sterically restricted configurational space, the docking algorithm is capable of finding only a single, most favorable pose. The remaining less favorable poses are characterized by significantly higher energy levels (at least 4 kJ/mol in reference to the lowest-energy pose). Thus, they were not considered in the subsequent analysis.

The position of OA in the enzyme cavity allows blocking of the catalytic site. In particular, the ligand molecule is arranged in close proximity to the catalytic His 440 residues. This steric hindrance is further supported by restricting the approach of potential substrates to the catalytic site. This can be attributed to the positioning of the ligand molecule within the vestibule of the binding site. The central fragment of the OA molecule, consisting of aliphatic and cyclic segments, remains in contact with a large aromatic cluster formed by numerous sidechains of the following residues: His440, Phe288, Phe290, Phe330, Phe331, Tyr121, Tyr334, Trp84, and Trp279. These ligand–AChE contacts can be categorized as CH-π interactions. These types of interactions are possible to occur between the aromatic and alkyl moieties; in the present case, the aromatic contribution always comes from the AChE residues (as listed above). The CH-π interactions have been identified as one of the essential driving forces playing a role in ligand–protein binding [13,14,15], particularly when a ligand molecule of aromatic character interacts with a cluster of aliphatic amino-acid sidechain or the opposite. The two polar moieties of OA, i.e., carboxyl and hydroxyl groups, interact with more polar side chains located closer to the binding cavity entrance. For instance, the carboxyl moiety interacts with Asp72 and Ser122 as well as with the hydroxyl group of Tyr121, whereas the hydroxyl moiety exhibits contact with the backbone fragments of Gly335, Phe290, and Arg289. Some of these contacts occur via hydrogen binding. The close presence of some other residues, e.g., Ile287, seems to be an opportunistic consequence of the previously-listed, more intensive interactions. Thus, the main driving force for binding is the non-polar CH-π interactions, allowing for minimizing the solvent-accessible surface of the aromatic cluster within the binding cavity of AChE; the scarce polar interactions play only a supporting role. Such mechanism of binding is in line with great affinity for the lipid bilayer discussed in the previous subsection as well as with the large, experimental logP_ow_ value.

### 2.6. Inhibitory Effect of Oleanolic Acid on the SH-SY5Y Cell Viability

**Figure 5:**
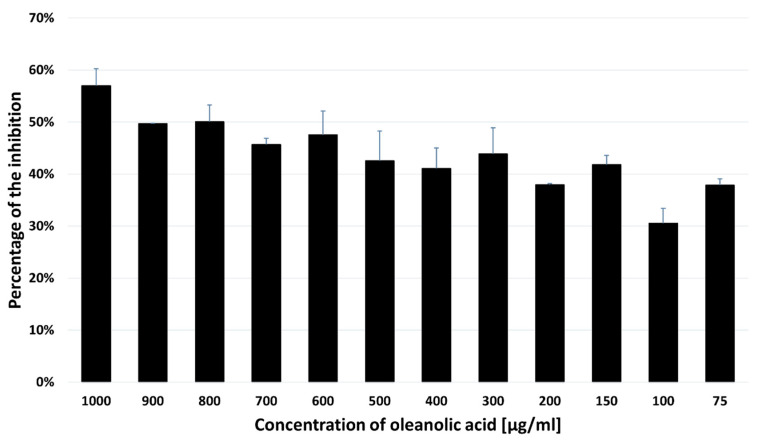
MTT results for the SH-SY5Y cells treated with OA. The OA IC_50_ value was calculated to be 715 µg/mL. The data are presented as the mean ± standard deviation (SD) of 3 independent experiments.

Effects of different concentrations of OA on the SH-SY5Y cells were measured using the MTT assays after 48 h of incubation (Figure 5).

### 2.7. Effect of Oleanolic Acid on the Cell Cycle Analyzed with the Flow Cytometry

**Figure 6:**
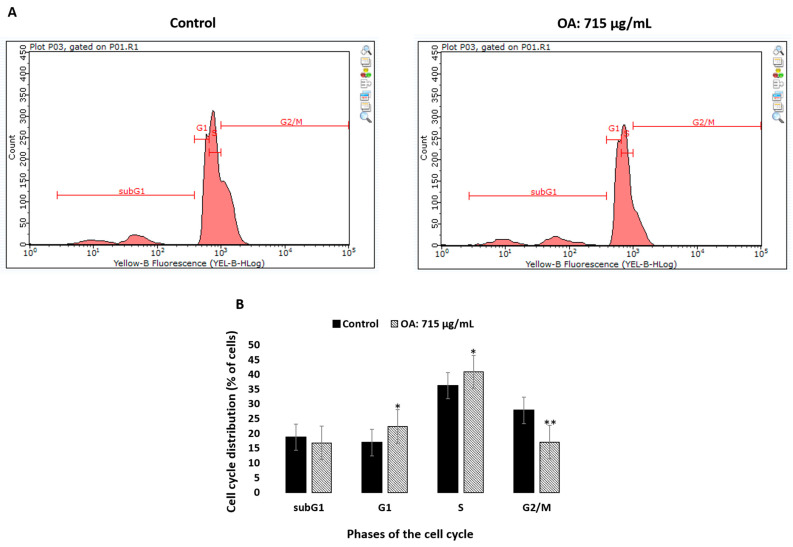
Effect on the phases of cell cycle in the SH-SY5Y cells treated with OA. (**A**)—The cell cycle distribution of PI-labelled cells was analyzed by flow cytometry in the SH-SY5Y control cells and cells treated with OA (715 µg/mL) for 48 h. (**B**)—The histogram shows the percentage of cells that are in different phases of the cell cycle. The results are expressed as the mean ± SD of three independent experiments. The *p* values below 0.05 (* *p* < 0.05, ** *p* < 0.0001) are considered statistically significant. The treated SH-SY5Y cells were arrested in the G1 phase and the percentage of cells in this phase increased from 16.87 ± 0.68% in the untreated cells to 23.57 ± 1.36% in the cells treated with oleanolic acid. Incubation with OA also caused an increase in the proportion of cells in the S phase (36.18 ± 1.50% vs. 40.85 ± 0.73%, *p* = 0.023) and a significant decrease in the G2/M phase (27.82 ± 0.86% vs. 17.02 ± 1.75%, *p* < 0.0001) of the cell cycle in the treated SH-SY5Y cells in comparison with the control.

In order to investigate whether OA treatment affected the cell cycle, the SH-SY5Y cells were treated with 715 µg/mL of the compound for 48 h, stained with PI, and subjected to the flow cytometry analysis. The effect on the cell cycle phases in the SH-SY5Y cells treated with OA is shown in Figure 6.

### 2.8. Oleanolic Acid Is Non-Toxic for Developing Zebrafish

**Figure 7:**
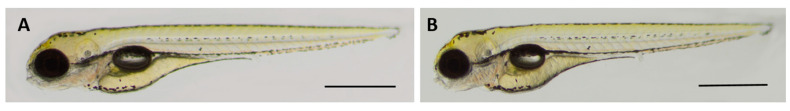
Representative photo of 4 dpf (**A**) control and (**B**) OA-treated larvae. Scale bars 1 mm.

The zebrafish embryos were incubated in 715 µg/mL of OA for 95 h, starting at 1 hpf. The representative photo of 4 dpf control and OA-treated larvae is shown in Figure 7. Our study proved that there was no difference between the control and experimental groups as regards the mortality rate in any of the analyzed time points (*p* > 0.05). Furthermore, the larval hatchability was not influenced by OA on 3- and 4-day-old fish (*p* > 0.05). Likewise, taking into account the obtained morphological parameters and the touch-evoked response assay, no difference between the tested groups (*p* < 0.05) was observed. The fish looked similar and reacted to touch, like their control counterparts (Figure 7). Therefore, one may conclude that at the dose of 715 µg/mL, OA is non-toxic in vivo for the developing organism—it does not influence mortality, hatchability, morphology, or muscle performance and its function.

## 3. Discussion

As mentioned above, neurodegenerative diseases with memory impairment constitute a growing problem mainly in aging societies. The World Health Organization predicts that the number of people suffering from dementia will be 132 million by 2050 [16]. Therefore, new therapeutic agents with possible reversal of memory impairment are constantly being sought. Undoubtedly, the plant kingdom is an interesting source of neuroactive substances, including neuroprotective and pro-cognitive ones.

The traditional Chinese herbs are undeniably a rich source of numerous substances acting on the CNS, e.g., *Ginkgo biloba* L. [17], *Panax ginseng* [18,19,20,21,22], or *Scutellaria baicalensis* [23,24,25,26]. Further, other plants grown in different parts of the earth are an invaluable source of neuroactive compounds, e.g., *Berberis integerrima* [27], *Carissa edulis* [28], *Melissa parviflora* [29], *Olea europaea* L. [30,31,32], *Salvia officinalis* L. [33,34,35,36], or *Rosmarinus officinalis* [37]. There are many chemical groups of CNS-active compounds: alkaloids [38,39], flavonoids [40,41], saponins [42,43], tannins [44,45], and terpenoids [46,47,48,49]. The mechanism of their action is based on the statement that they could affect both the CNS-nerve cells of the brain and the spinal cord, and thus, they can control, e.g., the mental activities, such as remembering or learning, and the autonomic nervous system, which is involved in the regulation of internal organs, heartbeat, circulation and breathing.

Oleanolic acid with numerous health-promoting properties [50] belongs to a group of pentacyclic triterpenoids whose, among others, cardioprotective [51,52,53,54], antioxidant [55], antimicrobial [56,57], anti-inflammatory [58], anticancer [59], pro-cognitive [60], hepatoprotective [61] properties have been proved by many scientific papers.

The in silico studies allowed the determination of the most important BBB-pharmacokinetic parameters, i.e., the distribution of a substance in the blood–brain area (logBB), the rate of passive diffusion/permeability (logPS), the brain/plasma equilibration rate (logPS_Fb_), the fraction unbound in plasma (Fu) and the fraction unbound in the brain (Fb). The blood–brain distribution (BB), frequently expressed as logBB, is defined as the ratio between the concentration in the brain and the concentration in the blood [62,63].

The in silico obtained logBB and Fu values (Table 1) indicate low brain penetration. However, it is proved that OA can affect the CNS, among others, by alleviating brain damage in ischemic stroke and other brain disorders [64] and can also be used as a potential therapeutic agent in the treatment of both the neurodegenerative and neuropsychiatric disorders [65,66]. Taking into account the permeability–surface area product (expressed as logPS) which is closely related to the cerebral blood flow (CBF), it can be stated that OA exhibited even a smaller BBB-permeability potential in comparison with other triterpenoids from the *Terminalia arjuna* bark, i.e., arjunic acid, arjunolic acid, arjunglucoside I, arjunetin, sericic acid, and arjungenin. The CBF ensures the proper delivery of oxygen necessary for the neuronal oxidative metabolism of energy substrates. The PS typically expressed in units of mL blood∕(100 g_tissue_·min), or mL blood (100 mL_tissue_·min) [67] or in mL blood/(h·kg) [68] is defined as the blood volume that flows per unit mass and per unit time in the brain tissue.

The smaller the values of the PS or Fb, the longer the time to reach the brain equilibrium is required. Therefore, the time to reach the brain equilibrium can be prolonged when the PS or the Fb decreases [68].

According to the free drug theory, each distribution process of a substance within a living organism depends on the unbound drug concentration [69,70]. As mentioned above, OA shows the Fu to be only 0.0055. Such a small value of the unbound fraction in plasma could indicate a partial or complete lack of ability to cross the BBB. Nevertheless, it is also hypothesized that drugs that strongly bind to plasma protein can rapidly dissociate and permeate through the BBB into the brain tissues [69].

Taking into account a function of the relative plasma and brain tissue unbound fractions at the distribution equilibrium (Kp, brain), it was observed that OA binds to the plasma proteins more extensively than those in the brain tissues. This lower CNS-distribution potential than those of the other triterpenoids from the *Terminalia arjuna* bark may result from significant impairment in the CNS distribution by, e.g., the efflux transport at the BBB [71]. Analyzing the value of the fraction unbound in plasma in the light of the physicochemical properties of the investigated molecule, it can be stated that Fu depends more on the lipophilic properties of the compounds than on their steric and electronic parameters. This is because the molecule is relatively small (MW less than 500 Da), and the topological polar surface area is less than 100 Å^2^. Numerous in silico studies, both earlier and contemporary, proved that there is a relationship between the physicochemical properties of a molecule and its BBB permeability [72,73,74,75,76,77]. The CNS drugs must cross the endothelium and partition into the aqueous environment of the cerebrospinal fluid and/or brain interstitial fluid [78]. Physicochemical parameters of a molecule, including steric, electronic, and lipophilic ones, are a key factor in this regard [79]. It is assumed that small hydrophilic compounds or those of large molecular weight (macromolecules), even if they are lipophilic, tend to be trapped in the cell membranes, and therefore they cannot cross the BBB passively [78]. The molecules of MW less than 400–500 Da can cross the BBB easily [80]. Besides the lipophilic and steric parameters, the topological polar surface area (TPSA) is of crucial importance for the ability of a substance to cross the BBB [81,82,83]. It is assumed that the substances that penetrate effectively into the brain tissues have a TPSA value of less than 100 Å^2^ or, even smaller, less than 60–70 Å^2^ [84].

The starting point for further research was the statement that among the physicochemical properties of a molecule, lipophilicity is of key importance for the ability to cross biological barriers. Thus, more realistic models for the determination of lipophilicity than the computational ones were applied, i.e., anisotropic membrane-like systems. Taking into account the obtained results (Table 2) of the logkw parameter considered as the lipophilicity descriptor [11], it can be seen that the logkw values are much lower than the logarithm of n-octanol/water partition coefficient determined in silico. This can result from the fact that n-octanol is an isotropic phase contrary to the investigated anisotropic membrane-like systems in which phospholipids are spatially ordered. Further, the electric charge of the phospholipid membrane, which is devoid of n-octanol, makes the membrane-like model more realistic [85].

Based on the Soczewiński–Wachtmeister equation [10], strong linear relationships between the logkw and s values were observed (Table 2) in all the tested membrane-like systems. It is known that higher s values indicate more lipophilic compounds. This is in accordance with the background retention theory where s values correspond to both solute/mobile phase and the solvent/stationary phase net interactions [10]. The obtained logkw values were used to determine the logBB ones based on our QSAR model. As can be seen in Figure 2, the calculated logBB values do not differ significantly. However, the value obtained from the cholesterol-bound column has a negative sign, unlike the other values. This can be due to the retention thermodynamics of OA caused by the lipophilic interactions between the solute and the cholesterol-bound stationary phase ligands, which contribute to the enthalpic changes in the stationary phase [86].

The results of the TLC-bioautography towards the AChE inhibitory potential of OA provided evidence that this property was dependent on the introduced concentration. Taking into account the AChE inhibitory potential of the other compounds of plant origin, it can be observed that OA exhibits a relatively strong inhibitory potential. The comparable IC_50_ values with that obtained for OA (9.22 μM) were determined for the following metabolites: infractopicrin (*Cortinarius infractus* Berk; Cortinariaceae; IC_50_ of 9.72 ± 0.19 μM), hydrohydrastinine (*Corydalis mucronifera* Maxim.; Papaveraceae IC_50_ of 9.13 ± 0.15 μM), jadwarine-A (*Delphinium denudatum*; Ranunculaceae; IC_50_ of 9.2 ± 0.12 µM), or coronaridine (*Ervatamia hainanensis* Tsiang; Apocynaceae; IC_50_ of8.6 μM) [6]. These compounds belong to the alkaloid chemical group. Among triterpenoids of the plant origin, there is 21β-Hydroxyserrat-14-en-3,16-dione from *Lycopodiella cernua* L. Lycopodiaceae) for which a similar IC_50_ value was observed (10.67 ± 0.66 µM) [6]. In comparison to galanthamine (IC_50_ of 3.52 µM [87]) being the first-line drug in the treatment of Alzheimer’s disease, it can be stated that OA is a promising compound in this regard.

The results of the AChE inhibition studies inspired the authors to investigate the molecular aspects of BBB permeation and the character of interactions with the AChE enzyme. Due to the fact that only a single compound is considered, we were not able to examine the correlation between the experimentally determined IC_50_ values and the theoretically predicted binding energies, as was performed in our previous study [87]. However, this value is in line with the expectation based on the large dimension of the considered molecule and its more hydrophobic character of OA in comparison to the other previously considered pentacyclic triterpenoid of plant origin, i.e., astragaloside IV [88].

The viability rate of SH-SY5Y cells was reduced to 71.5%, 61%, and 43% at the concentrations of 100 µg/mL, 300 µg/mL, and 1000 µg/mL, respectively, after 48 h of incubation with OA. Cytotoxicity of the compound against the SH-SY5Y cell line with the IC_50_ value was 714.32 ± 32.40 µg/mL. As shown in Figure 6, the treated SH-SY5Y cells were arrested in the G1 phase, and the percentage of cells in this phase increased from 16.87 ± 0.68% in the untreated cells to 23.57 ± 1.36% in the cells treated with OA. Incubation with OA also caused an increase in the proportion of cells in the S phase (36.18 ± 1.50% vs. 40.85 ± 0.73%, *p* = 0.023) and a significant decrease in the G2/M phase (27.82 ± 0.86% vs. 17.02 ± 1.75%, *p* < 0.0001) of the cell cycle in the treated SH-SY5Y cells in comparison with the control.

As regards the in vivo studies, there was no difference between the control and experimental groups as regards the mortality rate in any of the analyzed time points (*p* > 0.05). Furthermore, the larval hatchability was not influenced by OA on 3- and 4-day-old fish (*p* > 0.05). Likewise, taking into account the obtained morphological parameters and the touch-evoked response assay, no difference between the tested groups (*p* < 0.05) was observed. The fish looked similar and reacted to touch, like their control counterparts (Figure 7). Therefore, one may conclude that OA at the dose of 715 µg/mL is non-toxic in vivo for the developing organism—it does not influence mortality, hatchability, morphology, or muscle performance and its function.

## 4. Materials and Methods

### 4.1. Chemicals

The OA pharmacopoeial standard was purchased from Sigma Aldrich (Sigma Aldrich, St. Louis, MO, USA; p.a.). All the chromatographic measurements were made using acetonitrile (ACN; Sigma Aldrich, St. Louis, MO, USA; p.a)—water. Distilled water was obtained from the Direct-Q3 UV apparatus (Millipore, Warsaw, Poland). All solvents were >98% pure by the HPLC analysis.

### 4.2. In Silico Determination of Blood–Brain Barrier (BBB) Pharmacokinetic Descriptors

The BBB-pharmacokinetic descriptors were calculated using the ACD/Percepta software (version 2012, Advanced Chemistry Development, Inc., Toronto, ON, Canada).

### 4.3. Membrane-like Chromatographic Equipment and Conditions

The HPLC experiments were carried out on a Shimadzu Vp liquid chromatography system (Shimadzu, Kyoto, Japan) equipped with an LC 10AT pump, an SPD 10A UV-Vis detector, an SCL 10A system controller, a CTO-10 AS oven and a Rheodyne injector valve with a 20 µL loop. The solution of the pharmacopoeial OA standard was prepared in methanol (Merck, Darmstadt, Germany; p.a.) at a concentration of 1 mg/mL. The OA was found to be in the neutral form in solution under the experimental conditions. The optimization process was carried out before the chromatographic measurements. The flow rate of the mobile phases was set at 1 mL/min and the temperature at 20 °C. The analyte was detected with UV light at λ = 214 nm.

Three chromatographic columns, i.e., IAM.PC.DD2 column (IAM; 100 × 4.6 mm i.d., 10 µm; Regis Technologies, Morton Grove, IL, USA), cholesterol-bound (CHOL; Cosmosil; 75 × 2 mm i.d., 2.5 µm; Genore, Warsaw, Poland), and Internal Surface Reverse Phase (ISRP) were used as the anisotropic membrane-like stationary phases. The mobile phases were composed of acetonitrile-water as follows: 0.75; 0.80; 0.85; 0.90 *v/v* ACN-water for IAM, 0.70; 0.75; 0.80; 0.85 *v/v* ACN/water for CHOL, and 0.60; 0.65; 0.70; 0.75 *v/v* ACN/water for ISRP system. Each measurement was repeated three times.

The peaks of the citric acid were used as the dead time values. The average value of the obtained retention time was used to calculate the dead time for each system individually. The values of peak asymmetry factor were in the acceptable range.

### 4.4. Quantitative Structure-Activity Relationship (QSAR) Studies for Estimation of Permeation through the BBB

In order to examine the quantitative relationship between the ability of OA to cross the BBB and its physicochemical properties, the previously set up QSAR model was employed. The selection and multiple division of the dataset were thoroughly described in our previous paper [12]. Briefly, the establishment of a new model was based on the experimentally obtained distribution of the substance in the blood–brain area (logBB) values of 40 chemically diverse compounds [89]. The database was divided randomly into the test set and the training set (10 and 30 compounds, respectively). While setting up the model, the multiple linear regression (MLR) methodology with the backward elimination of variables was applied. This procedure was replicated several times to obtain the best fit between the logBB descriptor and the physicochemical parameters of OA. The predictive potency of the model was investigated based on the leave-ten-out (LTO) cross-validation. Moreover, the applicability domain was applied to evaluate the model’s reliability [12]. The following analyses of variance coefficients were performed, including the determination coefficient (R^2^), root-mean-square error (RMSE), root-mean-square error of leave-ten-out cross-validation (RMSECV), and predicted residual sum of squares (PRESS).

### 4.5. TLC-Bioautography Assay toward the Inhibition of Acetylcholinesterase (AChE) Activity

The OA pharmacopoeial standard purchased from Sigma Aldrich (St. Louis, MO, USA) was prepared at the concentration of 1 mg/mL in the double-distilled water: methanol (50:50 *v/v*), and it was applied on the aluminum sheet with the silica gel surface (10 cm × 10 cm TLC silica gel plate 60 F_254_, Merck, Darmstadt, Germany) with an autosampler (Camag, Muttenz, Switzerland). The reference solution was applied as 2, 4, 6, 8, and 10 µL volume bands. The measurement was repeated twice, and the average value of the obtained peak areas was taken for IC_50_ value calculation.

According to the previously published protocol [90], the enzymatic assay with some modifications was used, i.e., instead of chromatogram development, the TLC plate was directly sprayed with the substrate (2-naphtyl acetate) dissolved in distilled water with the quantity of 30 mg/20 mL.

The AChE enzyme (from the electric eel type VI-S, Sigma Aldrich, St. Louis, CA, USA) was dissolved in the aqueous solution of trizma buffer (pH 7.8) with bovine serum (500 mg/100 mL, Sigma Aldrich) with the quantity of 3 U/mL. The TLC plate previously dried in cold air was sprayed with the AChE solution and incubated at the temperature of 37 °C for the following 20 min in the humid incubator. After that, the Fast Blue B salt solution was prepared by dissolving the salt in distilled water to obtain a concentration of 0.612 mg/mL. Then, the TLC plate was sprayed with the Fast Blue B solution and visualized active zones as white spots against the violet background. The discolored areas corresponded to the AChE inhibitory activity of the respective zones.

Then, the dried TLC plate was analyzed using the Camag TLC visualizer in visible light. The peak areas of the discolored zones were automatically calculated by the WinCats software (v. 1.4, Camag, Muttenz, Switzerland), and their sizes were used to calculate the IC_50_ values that corresponded to the concentration of the standard providing half maximum inhibition of AChE enzyme.

### 4.6. Molecular Docking

The molecular docking was performed in accordance with the applied methodology and validated in our previous studies [88]. The OA molecule (deprotonated, as indicated by the corresponding pK_a_ value) was created using the online SMILES translator [91] and optimized within the UFF force field [92] (5000 steps, steepest descent algorithm) and the Avogadro 1.1.1 software [93]. The optimized ligand molecule was docked into a binding pocket of the protein structure found in the PDB database (PDB:1EVE) according to the flexible docking approach, i.e., the rotatable torsional angles in the ligand molecule were allowed to change their conformation. Docking was performed using the AutoDock Vina 1.1.2 software [94]. The process involved the 22 × 30 × 24 Å^3^ cuboid region, which includes the co-crystallized ligand present in the considered PDB entry as well as the nearest amino acid residues being in contact with this ligand. For efficient sampling of possible poses, the exhaustiveness parameter has been increased to 18, whereas the number of generated poses to 20. The exhaustiveness parameter affects the number of iterations in one run, and its increase decreases the probability of not finding the minimum binding energy. As indicated in systematic investigations, elevating the value of this parameter from the default one of 8 leads to results that are more realistic in the context of spatial variation of the docked ligands [95]. Furthermore, the docking procedure followed all standard protocols and algorithms set as default in AutoDock Vina. Alongside the ligand’s flexibility, rearrangements in the rotatable torsional angles were permitted for specific amino-acid side chains within the binding cavity. These included Tyr334, Phe288, His440, Gln74, Phe330, Phe75, Trp84, Glu199, Ser200, Tyr70, Tyr121, Trp279, Phe290, Phe331, Leu282, Trp432, Asn85, and Asp285. In order to assess the consistency of outcomes achieved through Vina’s inherently non-deterministic algorithm, the docking procedure was repeated three times.

### 4.7. Free Energy Profiles

Free energy profiles were calculated using the molecular dynamics (MD) approach along the 1D coordinate, which expresses the position of the OA molecule with respect to the homogeneous lipid bilayer. The methodology was analogous to that used in our previous work [88]. The MD simulations were conducted utilizing the GROMOS 53a6 force field [96] and the GROMACS 2016.1 MD package [97]. Each simulation system comprised the deprotonated OA molecule and a lipid bilayer. These components were placed within simulation boxes containing SPC water molecules [98], along with Na^+^ and Cl^−^ ions, to maintain a net charge of zero and an ionic strength of 0.15 M. Two versions of the lipid bilayer were considered: one composed exclusively of 1-palmitoyl-2-oleoyl-sn-glycero-3-phosphocholine (POPC) phospholipids and the other of 1-palmitoyl-2-oleoyl-sn-glycero-3-phosphoglycerol (POPG) phospholipids. The parameters for the phospholipids were sourced from [99], while those for OA were generated using the Automated Topology Builder online server [100]. Simulations were performed under periodic boundary conditions within rectangular computational boxes initially measuring around 8 × 8 × 12 nm. After geometry optimization and equilibration procedures, non-equilibrium pulling simulations were initiated in order to induce the migration of the OA molecule from the bulk solution through the lipid bilayer and back to the bulk solution. The harmonic potential associated with this process had a force constant of 5000 kJ mol^−1^ nm^−2^, and the pulling rate was set at 0.01 nm ps^−1^. The Z-axis position of the OA molecule perpendicular to the bilayer served as the pulling coordinate. The reaction coordinate was divided into 40 windows spanning approximately from −5 to 5 nm (with the center of the bilayer corresponding to zero), and 40 independent simulations were initiated. Each simulation employed an umbrella harmonic potential with a force constant of 1000 kJ mol^−1^ nm^−2^, based on the distance between the center of mass of the OA molecule and the box center. Data within each window were collected every 2 ps for a duration of 40 ns. After discarding the initial 5 ns for equilibration, 1D free energy profiles were constructed using the weighted histogram analysis method (WHAM) [101] implemented in GROMACS (as *gmx wham*) [102]. Statistical uncertainties in the energy profiles were determined using Bayesian bootstrapping of complete histograms [102]. Integration of equations of motion was carried out with a timestep of 2 fs. Bond lengths were constrained using the P-LINCS algorithm [103]. Temperature control (310 K) employed the V-rescale thermostat [104], while for pressure control (1 atm, semiisotropic coordinate scaling), the Parrinello–Rahman barostat with a relaxation time of 1 ps [105] was applied. Center of mass motion was removed at each step. Electrostatic interactions were treated using the particle-mesh Ewald (PME) method [106], with a short-range cutoff of 1.2 nm. Van der Waals interactions were gradually switched off at the distance from 1.0 nm to 1.2 nm.

### 4.8. Cytotoxicity Test in Human Neuroblastoma In Vitro

#### 4.8.1. Cell Culture

The human neuroblastoma SH-SY5Y cells (CRL-2266) obtained from American Type Culture Collection (Manassas, VA, USA) were cultured in the RPMI 1640 medium (Gibco, Invitrogen, Carlsbad, CA, USA) containing 8% FBS (Gibco, Invitrogen, USA), 5% penicillin and streptomycin (Gibco, Invitrogen, USA). At an initial plating density of 0.5 million cells per plate, cells were routinely trypsinized and subcultured.

#### 4.8.2. MTT Assay

The OA cytotoxicity against the SH-SY5Y neuroblastoma cells was determined using the methyl thiazol tetrazolium (MTT) assay. Cells were initially seeded on a 96-well plate with a density of 2 × 10^4^ cells per well and incubated in the CO_2_ incubator for 24 h at 37 °C. On the following day, the medium was replaced with a fresh medium supplemented with different concentrations of OA from 100–1000 µg/mL. Each 96-well plate was set up with control (untreated) and blank (medium only) groups. Three biological replicates with four technical replicates each were performed. After 48 h, the culture medium was replaced with fresh medium, and the cells were incubated with 20 µL of MTT working solution (Invitrogen, CA USA) (5 mg/µL) at 37 °C for 3 h. The supernatant was then removed, and purple formazan crystals were solubilized in 100 µL/well of dimethyl sulfoxide (DMSO), followed by incubation (30 min). The absorbance of the formazan solution was measured at 550 nm using a BioTek Epoch 2 Microplate Spectrophotometer (Agilent Technologies, Inc., Santa Clara, CA, USA), and the percentage of cell viability was calculated as follows: [viability (%) = (OD, experimental group − OD, control group)/OD, control group − OD, control group) × 100%]. The cell inhibition ratio (%) = 1 − the cell viability (%)]. The half maximal inhibitory concentration (IC_50_) was calculated by plotting the graph between the different concentrations of oleanolic acid and the percentage of cell growth inhibition.

#### 4.8.3. Cell Cycle Analysis

Propidium iodide (PI) was used to determine cell cycle progression. SH-SY5Y cells were seeded at 2 × 10^5^ cells/mL in 6-well plates and cultured for 24 h. Subsequently, the culture medium was replaced with the fresh medium (control cells), or cells were exposed to 715 µg/mL OA. Following 48 h incubation, the harvested cells were washed with PBS, centrifuged (300× *g*, 6 min, 4 °C), and fixed with ice-cold 70% ethanol at 4 °C overnight. According to the protocol of FxCycle™ PI/Rnase Staining Solution (Thermo Fisher, Scientific, Waltham, MA, USA), three wash steps with PBS were performed. The cells were resuspended to ~1 × 10^6^ cells. Analysis was performed using a Guava easyCyte flowcytometer (Merck, Germany). A total of 10,000 events were obtained per sample.

### 4.9. Toxicity Analysis in Zebrafish In Vivo

#### 4.9.1. Animals

The embryos of zebrafish (*Danio rerio*) that were used in the study were obtained from the Medical University of Lublin (Poland)–from the Experimental Medicine Centre. The animals were kept in an incubator in a specific zebrafish medium in the following conditions: at the temperature of 28.5 °C ± 0.5 °C, in the light/dark cycle of 14/10 h, and until the time of 96 h post-fertilization (hpf). The following study was conducted according to the National Institutes of Health Guide for the Care and Use of Laboratory Animals (Directive 2010/63/EU). The aforementioned directive states that there is no ethical approval needed for the yolk-feeding larvae that are up to 120 hpf. Nevertheless, during the experiment, all precautions were taken to limit the number of animals used in the study and to reduce their suffering. For euthanasia, a solution of 15 µM tricaine was used immediately after the completion of the planned experiment.

#### 4.9.2. Zebrafish Embryo Acute Toxicity Test

The OA potential toxicity (715 µg/mL) was examined in the zebrafish embryo acute toxicity test in accordance with the Organization for Economic Cooperation and Development (OECD) recommendation for the testing of chemicals (Test No. 236 [107,108]). Zebrafish embryos were screened for viability, shape, and transparency one hour after fertilization. Only the eggs meeting all criteria, i.e., being viable, completely transparent, and round, were chosen and moved to 12 well plates (Sarstedt, Germany). They were randomly divided into two groups, the control and experimental ones, and kept in each well until the time of experiment elapsed, i.e., up to 96 h. Two batches of embryos (3 wells per batch, n = 5–7 per well) were kept in 3000 µL of the zebrafish medium (control group) or supplemented with 715 µg/mL of OA (experimental group). The mortality rate was scored after 23, 47, 71, and 95 h of incubation. Hatchability was assessed after 71 and 95 h of incubation. The morphological abnormalities were assessed after 95 h of incubation: heartbeat, heart/yolk edema, yolk sac necrosis, hemorrhage [108], jaw development, eye size, and posture. Additionally, for assessing muscle function and performance, the touch-evoked response assay was conducted as described in detail previously [109,110].

#### 4.9.3. Statistical Analysis

The pooled data from the zebrafish experiment were analyzed using the Chi-squared or Fisher’s exact test (GraphPad Prism 9.3.1).

## 5. Conclusions

The results of the studies showed that oleanolic acid may be active towards the AChE enzyme. The in vitro and in vivo tests confirmed its safety in the used doses. Therefore, this can be a novel drug candidate with neuroprotective potential in the treatment of neurodegenerative diseases and an interesting source of further, more profound research in this aspect.

## Figures and Tables

**Table 1:** BBB-pharmacokinetic and physicochemical parameters of OA obtained in silico (ACD/Percepta software).

logBB in silico	logPS	logPS_Fb_	Fu	Fb	logPow	TPSA [Å^2^]	MW [g/mol]
−0.45	−4.3	−6.1	0.0055	0.02	11.108	57.53	456.7

**Table 2:** The parameters of the Soczewiński–Wachtmeister equation calculated for the chromatographic systems under investigation.

Membrane-like System	logkw	s	R^2^
IAM	1.656	1.515	0.991
CHOL	2.361	2.037	0.993
ISRP	0.637	1.853	0.983

**Table 3:** Parameters for determination of logBB value using the QSAR analysis.

Membrane-like System	Logkw	logPcw	ΔlogP	E
ISRP	0.637	8.995	8.358	1.46
IAM	1.656		7.339	
CHOL	2.361		6.634	

## Data Availability

Data is contained within the article.
